# Comparison of the Diagnostic Performances of Five Different Tests in Diagnosing Visceral Leishmaniasis in an Endemic Region of Ethiopia

**DOI:** 10.3390/diagnostics14020163

**Published:** 2024-01-11

**Authors:** Dawit Gebreegziabiher Hagos, Yazezew Kebede Kiros, Mahmud Abdulkader, Henk D. F. H. Schallig, Dawit Wolday

**Affiliations:** 1 Department of Medical Microbiology and Immunology, College of Health Sciences, School of Medicine, Mekelle University, Mekelle 1871, Ethiopia; dawitg@mu.edu.et (D.G.H.); dawwol@gmail.com (D.W.); 2 Laboratory for Experimental Parasitology, Department of Medical Microbiology and Infection Prevention, Amsterdam University Medical Center, Meibergdreef 9, 1105 AZ Amsterdam, The Netherlands; 3 Infectious Diseases Programme, Amsterdam Institute for Infection and Immunity, 1105 AZ Amsterdam, The Netherlands; 4 Department of Internal Medicine, College of Health Sciences, School of Medicine, Mekelle University, Mekelle 1871, Ethiopia; yazezew@gmail.com; 5 Department of Biochemistry and Biomedical Sciences, McMaster University, Hamilton, ON L8S 4K1, Canada

**Keywords:** rk39 rapid diagnostic test, direct agglutination test, microscopy, loop-mediated isothermal amplification, miniature direct-on-blood polymerase chain reaction–nucleic acid lateral flow immunoassay, diagnostics, Ethiopia

## Abstract

The lack of accurate and feasible diagnostic tests poses a significant challenge to visceral leishmaniasis (VL) healthcare services in endemic areas. To date, various VL diagnostic tests have been or are being developed, and their diagnostic performances need to be assessed. In the present study, the diagnostic performances of rk39 RDT, the direct agglutination test (DAT), microscopy, loop-mediated isothermal amplification (LAMP), and miniature direct-on-blood polymerase chain reaction–nucleic acid lateral flow immunoassay (mini-dbPCR-NALFIA) were assessed using quantitative polymerase chain reaction (qPCR) as the reference test in an endemic region of Ethiopia. In this study, 235 suspected VL cases and 104 non-endemic healthy controls (NEHCs) were recruited. Among the suspected VL cases, 144 (61.28%) tested positive with qPCR. The sensitivities for rk39 RDT, DAT, microscopy, LAMP assay, and mini-dbPCR-NALFIA were 88.11%, 96.50%, 76.58%, 94.33%, and 95.80%, respectively. The specificities were 83.33%, 97.96%, 100%, 97.38%, and 98.92% for rk39 RDT, DAT, microscopy, LAMP assay, and mini-dbPCR-NALFIA, respectively. In conclusion, rk39 RDT and microscopy exhibited lower sensitivities, while DAT demonstrated excellent performance. LAMP and mini-dbPCR-NALFIA showed excellent performances with feasibility for implementation in remote endemic areas, although the latter requires further evaluation in such regions.

## 1. Introduction

Millions of individuals across the globe are at risk of acquiring visceral leishmaniasis (VL), a deadly, neglected, (sub-)tropical infectious disease caused by parasites of the *Leishmania donovani* complex and transmitted through the bite of an infected female sand fly [1] belonging to the genus *Phlebotomus* in the old world or *Lutzomyia* in the new world [2,3]. Globally, approximately 50,000 deaths are recorded annually, and East Africa and Southeast Asia share the highest burden of the disease [4]. Over 90% of the world’s VL incidence is attributed to six less-developed countries, namely, Ethiopia, Bangladesh, India, Brazil, Sudan, and South Sudan [5,6]. VL, which is characterized by a protracted fever, fatigue, splenomegaly, anemia, and loss of weight, is lethal if the proper therapy is not given in a timely manner [7,8]. Post-Kala-Azar Dermal Leishmaniasis (PKDL), a macular, maculopapular, or nodular rash on the face, torso, or other parts of the body, is one of the complications of VL in immunocompromised individuals, which may occur some months after treatment [9,10]. Since the clinical presentations of VL mimic other febrile diseases and they often share the same geographic areas, the clinical features alone are insufficient to make a differential diagnosis and to prescribe anti-VL drugs. In addition, anti-VL drugs are not always safe and effective, and, therefore, empirical treatment is not recommended [11]. Consequently, rapid and accurate diagnostic tests are critical to start the best possible treatment options.

In most VL-endemic, resource-limited countries, the diagnosis of VL relies on a combination of the patient’s clinical presentation and rapid serological tests and/or microscopy of Giemsa-stained tissue aspirates [12,13,14]. Despite its poor performance and risky specimen collection procedures, microscopy is often still used as the reference standard for VL diagnoses in many endemic countries. Although serologic tests, primarily the rk39-based rapid diagnostic test (RDT), are highly variable and have moderate diagnostic performance, they are frequently used as first-line screening tests [15,16,17]. The direct agglutination test (DAT) has better sensitivity and specificity than rk39 RDT, but it requires an overnight incubation and more laboratory infrastructure, and it is used as an alternative sero-diagnostic test [18,19]. The main drawback of any of the available serological tests is their inability to differentiate between active VL infections from past or asymptomatic infections, especially in endemic areas [19].

Molecular tests, like end-point polymerase chain reaction (PCR) and quantitative PCR (qPCR), are highly sensitive and specific tests [20,21]. However, due to their time-consuming procedures, costly equipment and reagents, and infrastructural requirements, the implementation of molecular tests is hindered in resource-limited countries [22]. In response to this, nowadays, efforts are being made to innovate and develop sensitive, specific, and rapid tests that are feasible to implement in remote VL-endemic areas in resource-constrained countries [23,24,25]. Therefore, the discovery of cost-effective and user-friendly tests is crucial to improve the diagnosis and monitoring of VL, particularly in laboratories with limited resources [26]. The loop-mediated isothermal amplification (LAMP) [27,28,29] assay and miniature direct-on-blood polymerase chain reaction combined with nucleic acid lateral flow immunoassay (mini-dbPCR-NALFIA) [30] are two of the most effective diagnostic techniques that can be used in resource-limited countries.

The LAMP assay is a rapid (1 h until a result), sensitive, and specific molecular diagnostic assay that can amplify DNA at a constant temperature without the need for an expensive thermocycler [31,32]. The LAMP assay is based on auto-cycling and a highly efficient DNA denaturing process mediated by *Bst* DNA polymerase [33]. The amplification processes of the LAMP assay take place in two repeated steps: steam loop structure formation and auto-elongation at the 3′ end of the template gene and consecutive annealing and growing of primers using the loop region as the template at an isothermal temperature [27]. It has been successfully used for detecting *Leishmania* parasites, and the assay has a proven potential for the diagnosis of VL in resource-constrained areas [34].

Similarly, the mini-dbPCR-NALFIA is a low-cost and simplified molecular test that combines target DNA amplification directly from blood samples using a very simplified and portable mini-PCR machine and a nucleic acid lateral flow immunoassay detection system [35]. The assay utilizes a portable thermocycler that can be operated using a portable power supply and solar panels, making it useful in areas where electricity is not available or reliable [30]. Additionally, the mini-dbPCR-NALFIA test was developed to circumvent DNA extraction, reducing the risk of contamination during that process [30]. Moreover, the mini-dbPCR-NALFIA uses a smartphone application to control the lysis and amplification process [30]. The resulting readout from the NALFIA strip is easy to interpret, similar to rapid dipstick serological tests, and does not require any specialized skills or equipment. The results of LAMP and mini-dbPCR-NALFIA tests are read by visual inspection.

The research presented in this paper was designed to compare the diagnostic performances of four different diagnostics, i.e., rk39 RDT, DAT, LAMP assay, and mini-dbPCR-NALFIA, using qPCR as a reference standard for the diagnosis of VL in the Tigray region, north Ethiopia. We also included data from a microscopic examination of Giemsa-stained splenic or bone marrow tissue aspirates as a confirmatory test. However, this set of data is not complete as, in several cases, the patients were ineligible to undergo the sample collection procedure. These criteria include hemoglobin level, platelet count, age, and other underlying medical conditions. The Tigray region is highly endemic for VL, and there is a pressing need to implement novel diagnostic tests in (remote) health centers in this region. This study included HIV-positive as well as HIV-negative cases. This is of particular interest as HIV-positive cases tend to have a lower immune response and might be false negative in serological assays [36].

## 2. Materials and Methods

### 2.1. Ethical Considerations

Ethical clearance was obtained from Mekelle University, College of Health Sciences, and Ayder Comprehensive Specialized Referral Hospital, Health Research Ethics Review Committee (ERC number: 1102/2017), Mekelle, Tigray, north Ethiopia. Informed consent or assent was also obtained from the study participants or their guardians. In addition, a permission letter was obtained from the local authorities for each study site.

### 2.2. Study Site and Participants

A prospective cohort was established between July 2019 and October 2020 at Ayder Comprehensive Specialized Referral Hospital, Mekelle University, College of Health Sciences, Mekelle, Tigray, north Ethiopia. The objective of this study was to compare the diagnostic performances of 5 different VL diagnostic tests: rk39 RDT, DAT, microscopy, LAMP, and mini-dbPCR-NALFIA with qPCR as a reference standard. Cases with the clinical suspicion of being VL patients were prospectively enrolled, and clinical data and blood samples for diagnostic procedures were collected. The HIV status of all suspected VL cases was known prior to enrollment. Patients who had a VL treatment history within the past three months from enrollment and/or were unable to understand and/or those who refused to give informed consent/assent were excluded from this study. All enrolled patients were first tested using the rK39 RDT and/or microscopic examination of Giemsa-stained splenic or bone marrow aspirates to detect *L. donovani* amastigotes.

Non-endemic healthy controls (NEHCs) were also included in this study. The NEHC participants were recruited from areas that have no history of previous reported VL cases and who had no travel history to a VL endemic area.

### 2.3. Laboratory Analysis of Blood Samples

Rk39 RDT: when VL-suspected patients visited the outpatient VL clinics in the study areas, they were screened using rk39 RDT (IT LEISH, Bio-Rad Laboratories. Inc, Lunteren, The Netherlands) according to the manufacturer’s instructions. The rk39 RDT cassette is coated with rk39 antigen at the bottom and with anti-rk39 antigen antibodies at the top of the strips [37]. When the strip was dipped into a well containing a mixture of one drop of buffer (50 µL) and 10–12 µL of whole blood, the mixture started to move up by capillary action. If the blood contained anti-*Leishmania* antibodies, a visible line developed in the test and control areas, and the blood was reported as positive, and if no line appeared in the test areas after 10 min of incubation, the blood was reported as negative [38]. A test result was considered to be invalid if a visible line did not develop in the control areas.

DAT: DAT was performed at the Academic Medical Centre of the University of Amsterdam, Department of Microbiology and Infection Prevention, Experimental Parasitology Laboratory (AMC), using the freeze-dried antigen (FD-Ag) produced by AMC. Dried blood spots (5 mm) of EDTA blood of study cases on Whatman filter paper were punched out and placed into the first column of the V-shaped micro-titer plate and immersed in 125 µL of normal saline [39]. The plates were incubated at ambient temperature (4–20 °C) for 18–24 h to elute antibodies [40]. A serial dilution was made by adding 50 µL dilution fluid (0.1 M β-mercapto-ethanol in 100 mL saline) into all columns, transferring 50 µL from the first column to the next column, and continuing until the 11th column [41]. The 12th column was used as a negative control and contained only the dilution fluid [1]. The DAT was considered positive if agglutination was observed at a titer of ≥1:3200 [39].

Microscopy: Microscopic examination was only performed for clinical purposes, and the data were collected from the patients’ medical charts. Microscopic examination was performed to detect the amastigote stage of *L. donovani* in Giemsa-stained splenic or bone marrow tissue aspirate at the Pathology Laboratory, Ayder Specialized Referral Hospital, College of Health Sciences, Mekelle University, and Kahsay Abera Hospital, Tigray, Ethiopia. The results were reported on a graded scale ranging from negative (grade 0) when no amastigotes were seen, to grade 5+, if 10–100 parasites per field under the 100× oil-immersion lens were observed. Moreover, grading was also used for reporting different parasite densities: grade 4+ for 1–10 parasites per field, grade 3+ for 1–10 parasites per 10 fields, grade 2+ for 1–10 parasites per 100 fields, and grade 1+ for 1–10 parasites per 1000 fields, all observed with a 100× oil-immersion lens [42].

LAMP assay: DNA was extracted from whole blood samples using the boil-and-spin method [43]. In short, 95 µL of a patient’s blood was mixed with 5 mL of 10% sodium dodecyl sulfate and mixed by inverting the mixture 10 times in a 1.8 mL Eppendorf tube. The mixture is next incubated for 10 min at room temperature and subsequently mixed to obtain a homogeneous solution. The mixture was subsequently incubated at 95 °C in a heat block after the addition of 400 µL of DNA-free water and spun down at 13,000 rpm for 3 min. The supernatant was collected for further analysis.

The LAMP assay was performed using the LoopAmp Leishmania kit as per the manufacturer’s instructions (Eiken Chemical Co., Tokyo, Japan) [44]. The kit uses 6 primers that target conserved *Leishmania* gene segments kDNA and 18S rRNA, respectively [13]. The LAMP assay utilized *Bst* polymerase, which has auto-DNA denaturing and amplification properties [33]. The LoopAmp kit included both positive and negative controls. For amplification, 3 µL of DNA extract was mixed with 27 µL of MQ-water, and the total run time was 40 min at 65 °C in the LF-160 incubator (HumaLoop M incubator, HUMAN, Wiesbaden, Germany). The results were visually inspected: positive samples turned a green color when exposed to UV light, while negative samples did not show any color change.

Mini-dbPCR-NALFIA: The mini-dbPCR-NALFIA technique used to diagnose VL detects of two distinct gene sets. The first set comprises the minicircle kinetoplast *Leishmania* DNA (kDNA) gene, which is highly conserved and present across all *Leishmania* species. The second gene is human glyceraldehyde 3-phosphate dehydrogenase (GAPDH), which serves as an internal amplification control. A mixture of 12.5 µL of Mytaq Blood PCR buffer (Meridian Bio-Sciences, Newtown, OH, USA), 8 µL of sterile water, and 0.625 µL each of forward and reverse primers (both at a final concentration of 250 nM) labeled with Dig.-DH and Bio.-DH (Eurogentec, Seraing, Belgium), respectively, were used for kDNA amplification, and 0.375µL each of forward and reverse primers labeled (both at a final concentration of 150 nM) with FAM-DH and Bio-DH (Eurogentec, Seraing, Belgium) were used for GAPDH amplification. The mini-dbPCR-NALFIA amplification involved the lysing of 2.5 µL of EDTA blood at 98 °C for 10 min using a mini16 PCR machine (Amplyus, Cambridge, MA, USA). The PCR tube strip was placed into the mini-PCR machine, which was connected to a smartphone app via Bluetooth to program the amplification process. The mini16 PCR amplification protocol was programmed as follows: initial denaturation at 95 °C for 3 s 1×, denaturation at 95 °C for 15 s 30×, annealing at 58 °C for 30 s 30×, extension at 72 °C for 45 s 30 cycles, and a final extension 72 °C for 120 s for 1×. The amplification process was also monitored with the mini16 mobile app (version: 2.0.5: 75e94f1) via Bluetooth.

After completion of the PCR cycle and briefly spinning down the reaction mixture, 10 µL of the amplification product was added into a 1.5 mL tube containing 140 µL of the PCRD flex buffer (Abingdon Health, York, UK). The mixture was gently vortexed, and the NALFIA strip was dipped in the solution. The incubation time of the strip in the mixture was 10 min, and the presence of 3 black lines (for kDNA, GAPDH, and flow control, respectively) was assessed by placing the strip on white tissue paper.

qPCR: Quantitative polymerase chain reaction (qPCR) was used as a reference standard to evaluate the different VL diagnostic tests. For qPCR, DNA was isolated from 50 µL of EDTA blood samples using automated extraction with a NucliSENS EMAG apparatus (bioMérieux, Marcy-l’Étoile, France). The isolated DNA was eluted in 25 µL EMAG elution buffer. Next, 1.25 µL of the DNA eluate was added to 11.25 µL of qPCR master mix, which comprised 6.25 µL of iTaq Universal Probes Master Mix, 0.325 µL of kDNA forward primer and kDNA reversed primer each (both at a concentration of 260 nM), 0.125 µL of kDNA probe (concentration 100 nM), 0.125 µL of forward and reverse GAPDH primer each (both at a concentration of 100 nM), 0.125 µL of GAPDH probe (at a concentration of 100 nM), and 3.85 µL of DNase-free water. The qPCR run was performed on a BioRad CFX96 Real-Time PCR Machine (BioRad Laboratories, Hercules, CA, USA), which was programmed as follows: UNG activation at 50 °C for 10 min, initial denaturation at 95 °C for 5 min, denaturation at 95 °C for 15 s, and annealing/elongation at 59 °C for 15 s. With each qPCR run, a 10-fold serially diluted standard curve of DNA extracted from cultured *L. donovani* promastigotes was used. The analysis of the qPCR results was performed using Bio-Rad Maestro data analysis software, version 2.3.

### 2.4. Statistical Analysis

Median with interquartile ranges (IQRs) were used to describe the continuous variables, whereas the categorical variables were expressed using proportions. The McNemar chi-square (χ^2^) test was used to assess if the diagnostic performance of the tests under evaluation significantly differed between HIV-positive and HIV-negative cases. The sensitivity, specificity, and positive and negative predictive values of the tests were determined using STATA Ver. 15.0, Stata Corp, College Station, TX, USA, using the results of qPCR as a reference standard. The agreement between each test with the reference standard and between each other was determined using Cohen’s kappa coefficient (kappa value). A *p*-value of less than 0.05% was considered statistically significant and the Standards for Reporting of Diagnostic Accuracy Studies (STARD) guidelines were followed for reporting the results.

## 3. Results

### 3.1. Study Participants

In total, 235 VL-suspected cases were recruited for this study. The median age of the VL-suspected participants was 24.0 ± 13.2 (SD) years. Approximately one-quarter of the study participants were pediatric patients (<18 years old). In total, 220 (93.2%) of the study participants were male, representing the majority, and 204 (86.8%) of them were from the Tigray region. The most common clinical presentations of the study participants were fever in 226 (96.2%) patients, splenomegaly in 179 (76.2%), weight loss in 218 (92.8%), and fatigue in 199 (84.6%), Table 1. In total, 175 (66.8%) of the study participants had abnormally high levels of aspartate aminotransferase (AST), and 101 (42.98%) had elevated alanine aminotransferase (ALT) enzyme levels. In total, 212 (90.2%) of the participants were anemic. Additionally, hepatomegaly was observed in only 73 (31.1%) participants, and elevated creatinine levels were detected in 23 (9.9%) participants.

### 3.2. Performance of the Tests

A total of 339 participants, comprising 235 VL-suspected cases and 104 NEHC participants, were enrolled in this study, and their blood samples were tested using five different VL diagnostic tests: rk39 RDT, DAT, LAMP assay, mini-dbPCR-NALFIA, and qPCR.

Due to the potential risk of splenic bleeding and a very painful bone marrow tissue collection procedure, a microscopic examination was performed for clinical purposes only. Therefore, microscopic examination was performed only in 142 (60.4%) of the VL-suspected patients. The sensitivity, specificity, and positive or negative predictive values of the five different VL diagnostic tests were computed against the reference standard (qPCR), as listed in Table 2. Using qPCR, 144 (61.28%) VL-suspected patients tested positive. For the purpose of comparison with the other five diagnostic tests, these cases were classified as confirmed VL patients because qPCR is the most sensitive test. Only one of the NEHC cases tested positive using qPCR.

One hundred fifty-eight (67.23%) of the VL-suspected participants tested positive with rk39 RDT. Furthermore, only 1 of the 104 NEHC was found to be positive with rk39 RDT (this was the same case that tested positive using qPCR). The overall rk39 RDT sensitivity and specificity were 88.11% (95% CI: 84.68–91.55%) and 83.33% (95% CI: 79.38–87.29%), respectively. The sensitivity of rk39 RDT in HIV-positive and VL-suspected patients was lower compared with HIV-negative and VL-suspected patients (86.9% vs. 95.2%). Similarly, the proportion of rk39 RDT positivity among HIV-positive patients was found to be significantly lower than among HIV-negative patients (McNemar’s χ^2^ test, *p* = 0.024).

DAT was performed on blood samples from 339 participants. In total, 138 of the 143 qPCR-positive patients were positive using DAT (titer ≥ 1:3200), resulting in a sensitivity of 96.50% (95% CI: 94.55–98.46%) and a specificity of 97.96% (95% CI: 96.45–99.46%). In total, nine patients had borderline DAT results (≥1:800 and <1:3200), and among these patients, three tested positive and six texted negative with qPCR. However, when the borderline results were considered as negative, the specificity of DAT increased to 99.47% (95% CI: 96.32–99.78%). Of the 182 DAT-negative (titer: <1:800) patients, two were found to be positive using qPCR tests. Similarly, among the patients who tested positive using qPCR, three had borderline results and two had negative results using DAT.

A microscopic examination of Giemsa-stained tissue aspirates was conducted on 135 VL-suspected patients, primarily for clinical purposes, and the data for this research purpose was collected from the patients’ medical charts. Of the total who had microscopic examinations, 85 (62.96%) were positive, and out of these patients, 32 (28.83%) and 42 (39.64%) of them were with grade 2 and grade 3 and above, respectively.

Notably, 27 (24.1%) specimens deemed negative using microscopic examination tested positive using qPCR. Importantly, none of the qPCR-negative samples were positive using microscopic examination. The comparative analysis of the average Cycle Threshold (Ct) values revealed that higher Ct values were found in microscopy-negative/qPCR-positive samples than in microscopy-positive/qPCR-positive samples. The overall sensitivity and specificity of the microscopic examination were 76.58% (95% CI: 69.43–83.72%) and 100%, respectively. Microscopy positivity was significantly higher in HIV-positive patients compared with HIV-negative patients (McNemar χ^2^ test, *p* = 0.001).

Additionally, there was an association between parasite density (grades) and the DAT titer. Specifically, 55 (71.43%) of the patients with a DAT titer ≥1:3200 exhibited grade 2 and above in microscopic examinations. All patients with negative microscopic results were also negative for DAT. However, 12 (24.49%) and 13 (26.53%) of the microscopy-negative patients had DAT results at the borderline and DAT-positive, respectively.

The LAMP assay had a sensitivity of 94.33% (95% CI: 91.84–96.81%) and a specificity of 97.38% (95% CI: 95.66–99.10%) using qPCR as a reference test. All HIV-VL co-infected patients tested positive using the LAMP assay, giving a sensitivity of 100%. However, the sensitivity was lower (89.2%; 95% CI: 84.9–93.5%) in HIV-negative VL patients. On the other hand, the specificity of the LAMP assay among HIV-positive and HIV-negative patients remained almost the same (97.88% vs. 97.5%).

The sensitivity and specificity of the mini-dbPCR-NALFIA to diagnose VL were 95.80% (95% CI: 93.10–98.50%) and 98.92% (97.54–100.31%), respectively, when using qPCR as a reference test. Only one sample that was found to be positive using mini-dbPCR-NALFIA was negative using qPCR, and five samples that were positive using qPCR were negative using the mini-dbPCR-NALFIA. The sensitivity of the mini-dbPCR-NALFIA among the HIV-positive patients was higher (100%) than among the HIV-negative patients (91.7%, 95%CI: 73.25–98.12%). However, the specificity of the mini-dbPCR-NALFIA between HIV-positive and HIV-negative patients remained almost the same (99.5% vs. 100%).

The agreement of each diagnostic test with the reference standard was evaluated using Cohen’s kappa statistics. There was a strong agreement between qPCR and mini-dbPCR-NALFIA (k = 0.98, 95% CI: 0.82–1.00), as well as between qPCR and the LAMP assay (k = 0.96, 95% CI: 0.79–1.00). Similarly, the agreement between the DAT and qPCR also exhibited an excellent level of agreement (0.97, 95% CI: 0.84–1.01). However, the agreement between the rk39 RDT and qPCR was found to be lower (k = 0.85, 95% CI: 0.78–0.98) than that of the mini-dbPCR-NALFIA, LAMP assay, and DAT, but it was still excellent. In contrast, microscopic examination of Giemsa-stained tissue aspirates and the reference test qPCR showed only a substantial level of agreement (k = 0.80, 95% CI: 0.39–0.69). Additionally, the agreement between, respectively, the LAMP assay and mini-dbPCR-NALFIA (0.96, 95% CI: 0.86–1.00), the LAMP assay and microscopy (0.79, 95% CI: 0.65–0.95), the LAMP assay and DAT (0.95, 95% CI: 0.76–0.99), the LAMP assay and rk39RDT (0.85, 95% CI: 0.74–0.94), the mini-dbPCR-NALFIA and DAT (0.97, 95% CI: 0.85–1.01), the mini-dbPCR-NALFIA and rk39 RDT (0.85, 95% CI: 0.75–0.97), the mini-dbPCR-NALFIA and microscopy (0.81, 95% CI: 0.71–0.91), DAT and rk39 RDT (85, 95% CI: 0.69–0.98), and DAT and microscopy (0.82, 95% CI: 0.72–0.93) was assessed. All agreements except for the agreement between the LAMP assay and microscopy and rk39 RDT and microscopy were excellent.

## 4. Discussion

Accurate and prompt diagnosis of VL is essential to timely start appropriate treatment and to ensure proper case management [45]. However, in many VL endemic countries, due to restraints in resources, the implementation of accurate, affordable, and robust diagnostic tests remains a major challenge [46]. Currently, several diagnostic tests are used for the diagnosis of VL, each with varying degrees of diagnostic accuracy [47]. The present study was designed to evaluate the performances of five different VL diagnostic tests that require (relatively) little infrastructure against qPCR as a reference standard.

The reference test, qPCR, identified 144 out of the suspected patients (61.28%) as VL cases, and only one participant tested positive in the healthy control group. Thus, 91 (38.72%) cases among the clinically suspected cases were ruled out with the highly sensitive reference test, qPCR. This underscored that relying solely on the clinical case definition is insufficient for distinguishing VL cases from other diseases with similar symptoms. It also emphasized the need for the implementation of rapid, robust, and straightforward laboratory tests in close proximity to communities in need. The sensitivity of the diagnostic tests under evaluation varied, from the lowest sensitivity observed for microscopic examination of Giemsa-stained tissue aspirates (75.89%) and rK39 RDT (88.11%) to the highest value for DAT (96.50%) and the two simple molecular tests, the LAMP assay (94.33%) and mini-dbPCR-NALFIA (95.80%).

The overall sensitivity of rk39 RDT was 88.11% in the present study, which was consistent with our previous meta-analysis study performed in Ethiopia [48]. In addition, similar results were reported from different studies conducted in Ethiopia [49]. However, our findings indicate that the sensitivity was slightly lower than those presented in the global reports but higher than those observed in East African studies [18,19]. Parasite heterogeneity particularly in the kinesin gene [50], variation in study populations [51], variation in the reference standard used, and commercial brand differences were the main reasons for the performance variation [52,53,54,55]. This study also revealed that the sensitivity of rk39 RDT in HIV-positive VL patients was lower (86.9%) than in HIV-negative cases (95.2%), which is consistent with a number of studies performed in different parts of the world [1,18,48]. The differences could be explained by the impact of the virus as well as the *Leishmania* parasite, both compromising the immune response (antibody production), which is the main analyte of the rk39 RDT [1,48].

DAT was performed with a positive cut-off titer of ≥1:3200, a negative titer of ≤1:800, and a borderline titer of 1:1600. The overall sensitivity of DAT was 96.5%, which is significantly higher than the values observed in some studies conducted in Ethiopia [1], and slightly higher than those found in previous studies conducted in Africa [18,43,51,56]. However, these studies used microscopy as a reference standard, which is less sensitive than the DAT. Similar to rk39 RDT, the sensitivity of DAT was lower in HIV-positive cases compared with HIV-negative cases, which is in agreement with previous reports in Ethiopia [1,48,57]. Nevertheless, there was less variability observed in the specificity of DAT across the various reports.

Microscopic examination of Giemsa-stained tissue aspirates is commonly considered as the gold standard test, despite its inherent risk and discomfort. Previous research has consistently indicated that this method exhibits a notably low and variable sensitivity. Our findings were in agreement with these previous reports [49,58], revealing that the sensitivity of the microscopic examination of Giemsa-stained tissue aspirates is lower than any of the other five tests.

In contrast to serology, microscopy exhibited higher sensitivity in individuals who were HIV-positive in comparison with those who were HIV-negative. This phenomenon could be attributed to compromised immune systems in HIV-positive patients, resulting in elevated parasite loads in the tissue aspirate and subsequently, a higher likelihood of a positive microscopic examination [59]. Furthermore, we observed a significant association between parasite density and DAT titer, which is in agreement with a study performed in Sudan [60], As the density of parasites in the microscopic examination increased, there was a corresponding increase in antibody titer and probability of testing positive for DAT [60]. This indicates the interplay between the density of parasites and the test outcomes, reinforcing the importance of parasite load in diagnostic accuracy.

The LAMP assay and mini-dbPCR-NALFIA are recently developed Point-of-Care (PoC) tests for the diagnosis of VL, and they are primarily designed to be implemented as molecular diagnostic tests in remote and/or resource-limited endemic areas. The sensitivity and specificity of the LAMP assay were 94.33% and 97.88%, respectively. In addition, the boil-and-spin DNA extraction method is another advantage of LAMP because it is very easy to perform and its efficacy is comparable with the Qiagen DNA extraction method [44].

The mini-dbPCR-NALFIA is another simple, robust, and rapid test that combines molecular and immunological techniques. In the current study, the mini-dbPCR-NALFIA exhibited comparable performance to the LAMP assay and DAT, but it demonstrated significantly superior performance when compared with microscopy and rk39 RDT. In addition to its robust performance, the mini-dbPCR-NALFIA has been adequately simplified, making it suitable for implementation in remote endemic areas. It also meets the WHO-recommended ASSURED criteria for PoC tests. Due to its novelty, apart from our team’s earlier laboratory development and evaluation, so far, there have been no studies performed to further confirm the diagnostic performance of the mini-dbPCR-NALFIA.

## 5. Conclusions

To conclude, the rk39 RDT and microscopic examination of Giemsa-stained tissue aspirates exhibited lower sensitivity compared with the DAT, LAMP assay, and mini-dbPCR-NALFIA examinations. Compared with microscopy, rk39 RDT demonstrated superior sensitivity but lower specificity. DAT demonstrated high diagnostic performance, which was comparable to the LAMP assay and mini-dbPCR-NALFIA.

The LAMP assay and mini-dbPCR-NALFIA demonstrated excellent diagnostic performance and are rapid, simple, and feasible to deploy in endemic areas. Therefore, we recommend the continued use of rk39 and DAT in areas where needed, the substitution of microscopy with the rapid, robust, and feasible molecular test, the LAMP assay, and suggest further on-site investigations into the mini-dbPCR-NALFIA.

## Figures and Tables

**Table 1:** Clinical and socio-demographic characteristics of the VL-suspected study participants.

Characteristic	All VL Cases (*n* = 235)	qPCR+ (*n* = 144/235)	rk39 RDT+ (*n* = 158/235)	DAT+ (*n* = 141/235)	Microscopic+ (*n* = 85/135)	LAMP+ (*n* = 135/226)	dbPCR-NALFIA+ (*n* = 138/235)
Sex							
Male	220 (93.6%)	142 (60.4%)	155 (66.0%)	139 (59.1%)	84 (62.2%)	131 (95.0%)	132(95.73%)
Female	15 (6.4%)	2 (0.85%)	3 (1.80%)	2 (0.85%)	1 (0.74%)	4 (1.77%)	6 (4.3%)
Signs and symptoms							
Fever (>2 wks)	226 (96.2%),	139 (96.5%)	148 (93.7%)	133 (95.7%)	83 (98.8%)	128 (94.8%)	132 (95.7%)
Wt. lost	218 (92.8%)	137 (95.1%)	139 (87.9%)	134 (95.0%)	79 (92.9%)	126 (93.3%)	130 (94.2%)
Fatigue	199 (84.6%).	123 (85.4%)	132 (83.5)	126 (89.4%)	82 (96.5%)	128 (94.8%)	131 (94.9%)
Abdominal swelling		112 (77.8%)	101 (65.2%)	108 (76.6%)	72 (84.7%)	103 (76.3%)	109 (78.9%)
Splenomegaly	179 (76.2%),	134 (93.1%)	118 (74.7%)	125 (88.7%)	77 (90.6%)	129 (95.6%)	111 (80.4%)
Hepatomegaly	73 (31.1%)	64 (44.4%)	41 (25.9%)	48 (34.0%)	33 (38.8%)	42 (31.1%)	51 (37.0%)
Laboratory findings							
WBCs (×10^9^/L)	1.9 (1.5–2.3)	1.7 (1.1–2.6)	1.8 (1.3–2.1)	2.0 (1.6–2.3)	1.7 (1.1–1.9)	1.6 (1.2–2.2)	1.65 (1.2–2.1)
Hemoglobin (mg/dL)	7.6 (6.3–11.3)	7.8 (6.3–12.0)	7.9 (6.6–10.1)	8.1 (6.8–12.0)	7.2 (6.1–9.4)	7.8 (6.5–10.2)	7.4 (5.9–9.9)
Platelet count (3109/L)	172 (71–352)	167 (165–345)	177 (154–243)	171 (129–271)	169 (116–237)	174 (124–299)	169 (153–253)
AST(U/L)	175 (66.8%)	68 (43–168)	71 (39–173)	66 (45–171)	70 (44–143)	65 (39–169)	66 (40–155)
ALT(U/L)	41 (23–65)	42 (21–63)	41 (27–67)	39 (28–65)	38 (26–59)	42 (27–61)	39 (24–67)
Alkaline phosphate (U/L)	166 (81–342)	163 (78–298)	171 (77–355)	162 (69–343)	159 (89–236)	164 (81–309)	163 (79–321)
Creatinine (mg/dL)	0.7 (0.48–0.9)	0.69 (0.46–0.87)	0.7 (0.5–0.97)	0.69 (0.5–0.87)	0.68 (0.5–0.87)	0.73 (0.5–1.01)	0.68 (0.47–0.96)

qPCR—quantitative polymerase chain reaction, rk39 RDT—rk39 rapid diagnostic tests, DAT—direct agglutination test, LAMP—loop-mediated isothermal amplification, dbPCR—NALFIA direct blood polymerase chain reaction–nucleic acid lateral flow immunoassay, Wt. lost—weight loss of the cases, AST—aspartate aminotransferase, ALT—alanine transaminase, and WBCs—white blood cells.

**Table 2:** The sensitivity, specificity, and negative and positive predictive values of the rk39 RDT, DAT, microscopy, LAMP assay, and mini-dbPCR-NALFIA tests used to diagnose VL in north Ethiopia compared with qPCR as a reference standard.

Tests	Sensitivity (95% CI)	Specificity (95% CI)	PPV (95% CI)	NPV (95% CI)
rk39 RDT	88.11% (84.68–91.55%)	83.33% 79.38–87.29%	79.25% (74.94–83.55%)	90.66% 87.57–93.75%
DAT	96.50% (94.55–98.46%)	97.96% (96.45–99.46%)	97.18% (95.42–98.94%)	97.46% (95.79–99.14%)
Microscopy	75.89% (68.68–83.11%)	100.00% (100.0–100.0%)	100.00% (100.0–100.0%)	46.00% (37.59–54.41%
LAMP assay	94.33% (91.84–96.81%)	97.88% (96.34–99.43%)	96.38% (94.37– 98.39%	95.88% (93.74–98.02%)
Mini-dbPCR-NALFIA	95.80% (93.68–97.93%)	98.99% (97.93–100.05%)	98.56% (97.30–99.83%)	97.03% (95.23–98.83%)

## Data Availability

The data presented in this study can be obtained by contacting the corresponding author with a reasonable request.
